# Supplementary material for: Femtosecond pulse amplification on a chip
Source: Nat Commun. 2024 Sep 16;15:8109. doi: 10.1038/s41467-024-52057-3 (PMC11405508; doi:10.1038/s41467-024-52057-3)
Supplement: Supplementary file 1 — Supplementary Information [file 41467_2024_52057_MOESM1_ESM.pdf]

Supplementary Information - Femtosecond pulse amplification on a chip

Mahmoud A. Gaafar[1,*], Markus Ludwig[1,*], Kai Wang[2], Thibault Wildi[1], Thibault Voumard [1], Milan Sinobad[1], Jan Lorenzen[1], Henry Francis[3], Jose Carreira[3], Shuangyou Zhang[4], Toby Bi[4,5], Pascal Del'Haye[4,5], Michael Geiselmann[3], Neetesh Singh[1], Franz X. Kärtner[1,6], Sonia M. Garcia-Blanco[2], Tobias Herr[1,6,**]

[1]Deutsches Elektronen-Synchrotron DESY, Notkestr. 85, 22607 Hamburg, Germany
[2]Integrated Optical Systems, MESA+ Institute for Nanotechnology, University of Twente, 7500AE, Enschede, The Netherlands
[3]LIGENTEC SA, EPFL Innovation Park, Chemin de la Dent-d'Oche 1B, Switzerland CH-1024 Ecublens, Switzerland
[4] Max-Planck Institute for the Science of Light, 91058 Erlangen, Staudtstr. 2, Germany
[5] Department of Physics, FAU Erlangen-Nürnberg, 91058 Erlangen, Germany
[6] Department of Physics, Universität Hamburg, Luruper Chaussee 149, 22761 Hamburg, Germany
[*]These authors contributed equally.
[**]tobias.herr@desy.de

1 Third order dispersion

For ultra-short pulses in the femtosecond regime, their large spectral bandwidth implies that third order dispersion effects become relevant. In our case, as evident in Fig. 3f in the main text, not perfectly compensated third order dispersion after compression leads to an asymmetric low intensity tail in the output pulse (and a 'butterfly' shape in the FROG trace). Complementing main text Fig. 2b, Figure S1 shows the third order dispersion in dependence of the silicon nitride waveguide width an the Al_2O_3 layer height. While the gain waveguides show close to zero third order dispersion, waveguide bends and tapers can exhibit considerable amounts of third order dispersion. Importantly, *both* signs of third order dispersion occur along the taper, so that they can compensate each other. In our case, although peak dispersion values exceeding 10 ps^3/km occur along the taper and during the bend, their mean value is much smaller and the different contributions approximately compensate each other (mean third order dispersion along a taper ca. 1.6 ps^3/km; mean dispersion in a bend ca. 7 ps^3/km; per straight gain waveguide the amplifier comprises ca. 2×3 mm of taper and a bend length of ca. 1.5 mm).

2 Amplification without pre-chirping

As mentioned in the main text, it is also possible to send the input pulses directly into the amplifier without pre-chirping. Due to the normal dispersion the pulses will spread in time before their gain in energy would cause substantial nonlinear effects. Figure S2 presents a numerical simulation of signal pulse amplification without pre-chirping the pulse, in the same representation as Fig. 4 in the main text). Figure S2a reveals the temporal dynamics of amplification and pulse broadening due to normal dispersion of the amplifier. To obtain short pulses, dispersion compensation would now be required after amplification, at higher power levels. In contrast to pre-chirping at low power, this is more challenging due to the non-trivial power-dependent nonlinear compression dynamics.

3 Critical power: $L_{\mathrm{NL}} = L_{\mathrm{D}}$

To provide an estimate of the maximally attainable peak power in a linear pulse propagation regime, we consider the critical power where nonlinear and dispersion length are equal $L_{\mathrm{NL}} = L_{\mathrm{D}}$. Figure S3 shows this critical power for different pulse duration (impacting L_{D}) and gain layer height (impacting L_{D} and L_{NL}), where we have assumed a fixed silicon nitride waveguide width of 300 nm. Figure S3 shows that a ~100 fs pulse can be amplified and compressed to its time bandwidth limit with peak power levels of approximately 900 W, while remaining in the linear pulse propagation

regime. For higher peak power levels nonlinearity will start contributing. Shorter pulses, which can in principle be supported by the Tm-doped gain medium could support higher peak power, in principle exceeding 10 kW, while remaining in the linear regime. To reach these power levels a careful management of higher order dispersion would be needed.

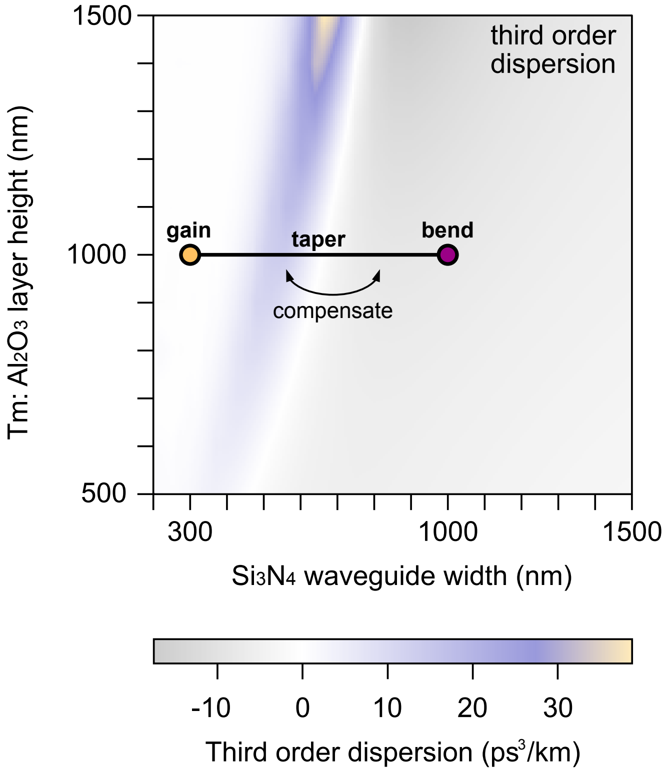

Figure S1 | **Third order dispersion** in dependence of the silicon nitride waveguide width an the Al_2O_3 layer height. Along the taper, both negative and positive third order dispersion are present and can compensate each other. Specifically, the taper profile may be chosen to obtain a net-zero third order dispersion amplifier.

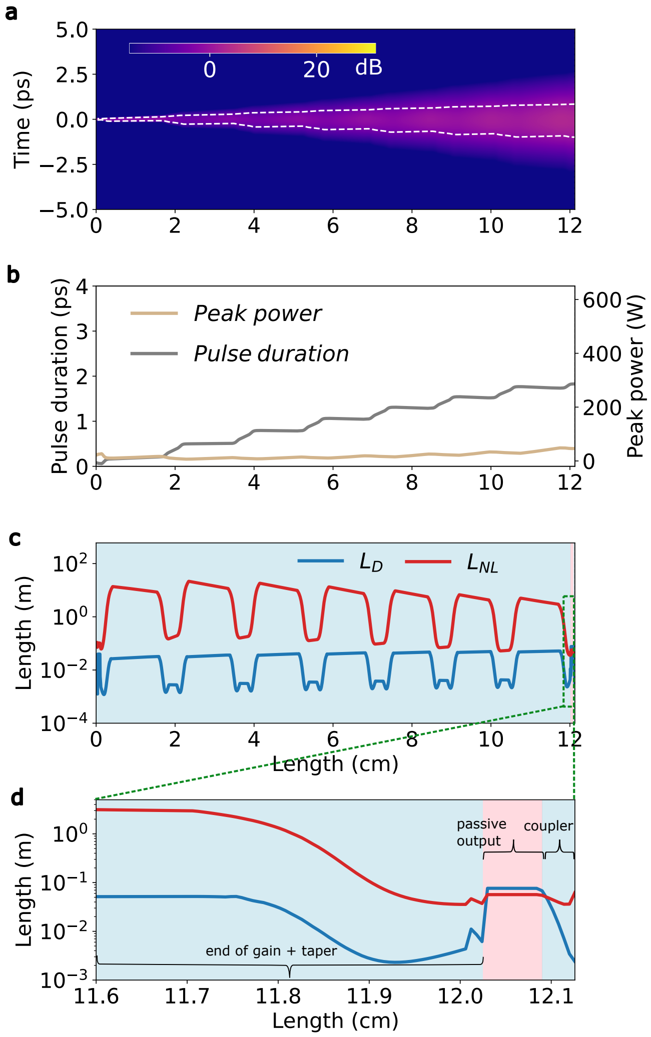

Figure S2 | Numerical simulation of signal amplification without pre-chirping. **a**, Evolution of the temporal pulse power while propagating through the amplifier chip in a co-moving reference frame. One sequence of the alternating gain, bend and taper sections is indicated. The contours indicate the full-width-half-maximum (FWHM) pulse duration. **b**, Pulse duration and pulse peak power while propagating through the amplifier chip. **c**, Evolution of the pulse's dispersion length L_{D} and nonlinear length L_{NL} while propagating through the amplifier chip. The blue background color highlights where the propagation is dominated by linear optical effects ($L_{\mathrm{D}} < L_{\mathrm{NL}}$); nonlinear optical effects dominate only in short section within the last 1.5 mm of the entire, $>$12 cm long propagation distance (red background color). **d**, shows a magnified portion of panel c., where the gain section and taper to the output waveguide, the passive output waveguide, and the inverse taper for chip-output coupling are indicated.

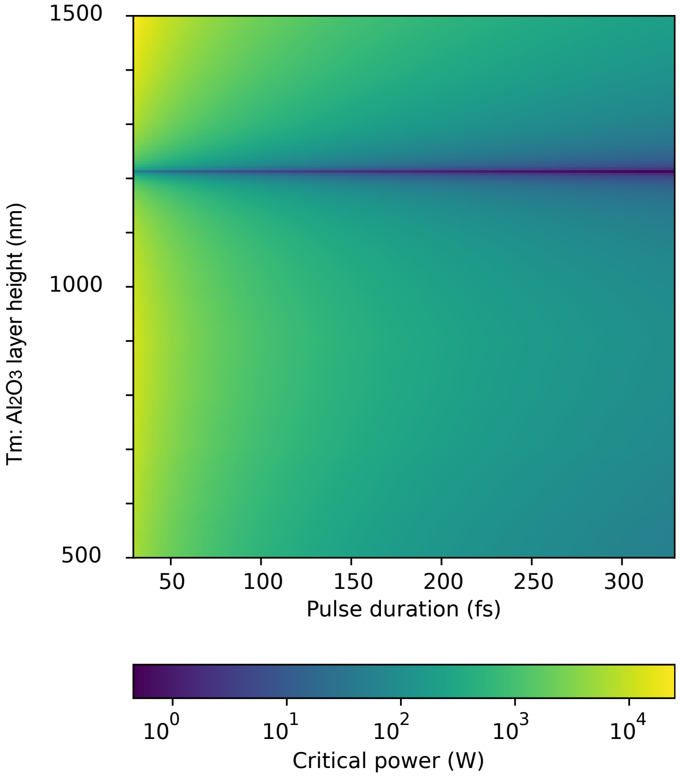

Figure S3 | **Critical power** in dependence of the pulse duration and the Al_2O_3 layer height (assuming 300 nm wide silicon nitride waveguide). The achievable peak power scales quadratically with the inverse pulse duration and linearly with the (absolute) value of the GVD. For a layer height of ~ 1200 nm, the GVD is close to zero and hence the critical power low. Higher critical power is achieved for shorter pulses and layer heights that exhibit larger GVD (cf. Fig. 2b in the main text).
